# Effects of water‐deficit stress and putrescine on performances, photosynthetic gas exchange, and chlorophyll fluorescence parameters of *Salvia officinalis* in two cutting times

**DOI:** 10.1002/fsn3.2741

**Published:** 2022-02-26

**Authors:** Maryam Mohammadi‐Cheraghabadi, Seyed Ali Mohammad Modarres‐Sanavy, Fatemeh Sefidkon, Ali Mokhtassi‐Bidgoli, Saeid Hazrati

**Affiliations:** ^1^ 41616 Department of Agronomy Faculty of Agriculture Tarbiat Modares University Tehran Iran; ^2^ 48498 Research Institute of Forests and Rangelands Tehran Iran; ^3^ 125619 Department of Agronomy Faculty of Agriculture Azarbaijan Shahid Madani University Tabriz Iran

**Keywords:** cutting, Pearson's correlation, potassium, stomatal conductance and sage

## Abstract

A 2‐year (2017–2018) field experiment was performed to specify if the foliar application of putrescine (PUT) under optimum and water‐deficit stress (WDS) conditions would favorably affect leaf gas exchange, greenness, chlorophyll fluorescence parameters, pigments, sodium (Na), potassium (K), as well as yield and content of the essential oil (EO) relationships in *Salvia officinalis* L. (sage) in spring (cutting 1) and summer (cutting 2). Based on the results analysis of variance, the effects of WDS, PUT, and cutting time were significant for the dry weight, leaf area index (LAI), EO content, EO yield, chlorophyll (Chl) t, carotenoid, Na, and K of sage. According to regression results, the response of EO content, EO yield, non‐photochemical quenching (NPQ), spad, Chl a, Chl t, K, and K/Na to WDS can be expressed by a quadratic model, indicating that they would attain their maximum in 75.5%, 34.86%, 38.33%, 84.13% 60%, 70%, 50.40%, and 40.28% available soil water depletion (ASWD), respectively. The response of dry weight, LAI, EO content, EO yield, Fv/Fm, spad, ΦpsII, Chl a, Chl b, Chl t, carotenoid, K, and K/Na to PUT can be expressed by a quadratic model, showing that they would attain their most under 0.98, 1.14, 1.34, 1.16, 1.27, 1.18, 1.17, 1.25, 1.17, 1.27, 1.31, 1.21, and 1.19 mM of PUT, respectively. These findings suggest that, probably, the functions and structures of the photosynthetic system were further enhanced with PUT, thereby they can be promoting primary electron transfer in PSII. Also, stomatal and photosynthetic activity improved with increasing K levels with PUT.

## Graphical abstract

A 2‐year experiment was conducted in water stress and putrescine (PUT) foliar application. Endogenous PUT, EO content, Pn, Gs, Na, and K were higher in spring than in summer. The response of Chl b & Fv/Fm to irrigation regime can be expressed by a linear model. Dry weight, LAI, EO yield, Fv/Fm, and NPQ were higher in summer than in spring. Putrescine and harvest time affected for Fv/Fm.

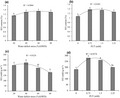

## 1 INTRODUCTION

Sage has the oldest history among medicinal plants. This plant belongs to the family Lamiaceae. The leaves of sage have been applied in pharmaceutical, perfumery, and food industries for centuries (Altindal et al., 2016). Secondary metabolites (SMs) indirectly affect the primary processes of metabolism and directly influence the plant adaptation to environmental stresses (Mohammadi et al., 2018; Verma & Shukla, 2015). SMs significantly ameliorate plant growth and development in several environmental factors, thereby acting as main primitive metabolites (Zandalinas et al., 2017). Water stress is the main ecological issue which leads to altered essential oil (EO) yield of *Cuminum cyminum* (Bettaieb et al., 2011). Also, water deficit stress (WDS) affects several physiological processes of plant including photosynthesis, respiration, transpiration, cell turgidity, stomatal conductance, and light absorption, finally resulting in reduced crop production (Hazrati et al., 2017). Photosynthetic processes in crops are largely affected by stresses, while photosynthesis is the most basic physiological process (Ashraf & Harris, 2013). There is evidence that WDS has a considerable effect on the performance of photosynthesis (Hazrati et al., 2016). The Chlorophyll (Chl) content of leaves is the important component of pigments that can straight affect the photosynthetic potential and so, initial production (Gitelson et al., 2003). Environmental stresses affect pigments and can prevent photosynthesis. Photosynthetic rate and Chl concentration change in plants under environmental stress (Ashraf & Harris, 2013). Many reports have indicated variations in the leaf Chl content under WDS. Indeed, pigments' concentration and structure are determinatives of light absorption efficiency (Anjum et al., 2011; Porcar‐Castell et al., 2014).

According to some reports, Chl concentration and carotenoid vary according to environmental conditions (Horton & Ruban, 2005). As reviewed by Müller et al. (2001), the light energy imbibed with Chl molecules can be managed in three ways: photosynthesis, heat, or fluorescence. The most important chlorophyll fluorescence parameters include non‐photochemical quenching (NPQ), Fv/Fm, photochemical quenching (qP), and ФPSII which are applied in plant stress physiology studies (Murchie & Lawson, 2013). According to many reports, photosynthetic efficiency of photosystem II (Fv/Fm) is the most widely applied chlorophyll fluorescence measuring parameter (Hazrati et al., 2016). As reviewed by Paknejad et al. (2007), PSII parameter indicates the quantum yield of noncyclic PSII photochemistry in plants that are stressed, and Fv/Fm is a fine indicator of light inhibition in stressed plants. Flow of electrons at PSII is indicated by this parameter (Moseki & Dintwe, 2011). NPQ parameter is the nonphotochemical quenching and is relevant to radiant energy loss as heat (Maxwell & Johnson, 2000). NPQ and qP would increase and decrease, respectively, under WDS (Ashraf & Harris, 2013). Also, for better understanding xanthophyll cycle activity, NPQ can be checked (Ralph & Gademann, 2005). Many physiological processes rely on cellular K, such as maintaining cellular turgor, regulating membrane permeability, regulating ion balance, enhancing photosynthesis, and influencing protein synthesis (Maathuis & Amtmann, 1999). The physical similarities between Na and K may cause Na to compete with K for entry into the symplast, resulting in a K deficiency. A high ratio of K to Na at binding places in the cytoplasm could inhibit enzyme functions and metabolic processes that rely on K (Maathuis & Amtmann, 1999). Other effective environmental factors include the seasons of year that influence the synthesis of chemical substances in some medicinal plants. Changes across different seasons may lead to corresponding shift in and/or accumulation of some EO compounds (Koptur, 1985). Souza et al. (2018) observed the volatile oil yield of seasonality in *Spiranthera odoratissima*. Zutic et al. (2014) indicated the significant effect of cutting time on the EO yield of sage. Several studies have been conducted on effective application of elicitors for the production of target SMs in plants (Emami Bistgani et al., 2017).

Polyamines (PAs) such as PUT, spermine, spermidine, and cadaverine as well as a class of phytohormone‐like aliphatic amine composites regulate many physiological processes such as development and growth, leaf senescence, plus abiotic and biotic plant stress reactions (Mohammadi et al., 2018). PAs contribute to the regulation of physical and chemical properties of membranes and nucleic acids (Aloisi et al., 2016). PAs can protect plants under abiotic stresses (Bouchereau et al., 1999). PAs can induce biosynthesis of secondary metabolites, increase permeability plus integrity of the plasma membrane, and inhibit the chlorosis under environmental stresses (Alcázar et al., 2010, 2011; Gupta et al., 2013; Kusano et al., 2007; Mohammadi et al., 2018). Some reports have indicated that PAs positively affected photosynthetic pigments in *Morus alba*, *Cucumber sativus*, and *Phaseolus vulgare* (He et al., 2002; Nassar et al., 2003).

However, the mechanisms of prohibition of photosynthesis by water deficit and PUT remain poorly defined. Hence, the current study was designed to check the role of exogenous (exo) PUT and WDS on leaf gas exchange, greenness index, chlorophyll fluorescence parameters, pigments, Na, K, EO content of sage at two cutting times.

## 2 MATERIALS AND METHODS

### 2.1 Position description and experimental design

1200 of sage seedlings was cultivated in Tarbiat Modares University's Agriculture Faculty located in Tehran, Iran (1200 m upper sea level and 35º 70´ N, 51º 40´ E), in 2 years (2017–2018). Thirty‐year mean annual temperature and rainfall are 22°C and 232.6 mm, respectively (see meteorological data in Supplementary Information 1). The experiments were as split–split plot adjustment in a randomized complete block design with three repetitions. The main plot was the water‐deficit stress (WDS), the subplot was PUT, and the sub‐subplot was the cutting time. The WDS was as follows: 20%, 40%, 60%, and 80% ASWD. WDS treatments were carried out based on the maximum allowable depletion (MAD) from the percentage of available soil water (%ASWD). Irrigation started after soil water reached a threshold level (Bahreininejad et al., 2013; Govahi et al., 2015). Time Domain Reflectometry probe (TDR) (Model TRIME‐FM, Germany) was used to measure soil water level at a root zone of sage (depth of 50 cm). Four concentrations of PUT (distilled water (0), 0.75, 1.5, and 2.25 mM) were used. All aboveground parts in each plant were exo sprayed 50 cm upper of plant. Foliar application of PUT was carried out twice each year, 1 week before using WDS in each cutting in 2 years. Furthermore, the foliage was handpicked in spring (cutting 1) and summer (cutting 2). No PUT and WDS were used in the first month of the growth cycle, because plants should have been formed of similar masses of foliage, before applying treatments. The cutting 1 and 2 carried out before flowering. The foliage of plants was cut 8–10 cm over the soil surface. Collections were carried out in 2 years (2017–2018).

### 2.2 Essential oil extraction

Dried samples (100 gr) were extracted via the hydrodistillation method in a Clevenger device with double‐distilled water (1000 ml). The collected surplus of aqueous EO was dried over anhydrous sodium sulfate. Then, the weight of pure EO was determined, and its percentage was computed.

### 2.3 Leaf gas exchange and leaf greenness quantification

An Li‐6400XT Portable Photosynthesis System (Li‐Cor Inc.) was utilized to determine the net photosynthetic rate (Pn) and stomatal conductance (gs) of plants per plot after each cutting time. The chamber was corrected to 25°C (temperature), ambient CO_2_ concentration (Ca) was 380 μM/mol, and photosynthetic photon flux density was 1250 μM/m^2^/s. For estimating the leaf greenness, GREENNESS INDEX‐502 was used on the same leaves mentioned previously (Konica Minolta).

### 2.4 Chlorophyll fluorescence parameters

The maximum performance of PSII (Fv/Fmand), nonphotochemical quenching (NPQ), photochemical quenching (qP) was determined applying a modulated fluorescence chlorophyll meter (MINI‐PAM, Walz). Leaves were dark‐adapted via leaf clips (15 min). After dark adaptation, the highest photochemical performance (Fv/Fm), actual photochemical performance (ΦPSII) = (Fm'‐Fs)/Fm', qP = (Fm'‐Ft)/(Fm'‐Fo'), and NPQ =1‐ (Fm'‐Fo')/(Fm‐Fo) were computed as well, and the light severity applied to calculate the yield of ΦPSII and NPQ was 1,200 μmol/m^2^/s (Roháček, 2002).

### 2.5 Photosynthetic pigments

To estimate the chlorophyll and carotenoid, the procedure of Lichtenthaler (1987) was used. Briefly, 0.5 g of fresh leaves sage was powdered applying mortar and pestle containing 10 ml of acetone (80% V/V). Then, the extract was centrifuged for 10 min (12,000 rpm). The light absorption was read at 645, 663, and 470 nm by a UV–vis spectrophotometer. Photosynthetic contents were expressed as mg/g/fw.

### 2.6 Endogenous putrescine analysis determination

Endogenous (endo) PUT extraction and thereafter HPLC measurement were performed following the procedure of Lütz et al. (2005). First, samples were injected into injector loop (20 µl) in RP‐C18 Column (15 cm × 4 mm i.d.), particle size (5 µm), at 30°C using Methanol: Water linear gradient from ratio of 50:50 to 80:20 (v/v) for 30 min. The last ratio was retained at 1 ml/min. PUT was detected by measuring the fluorescence intensity of samples (254 nm) and then comparing their peak times with those of standard PUT.

### 2.7 Estimation of Na^+^, K^+^, and leaf area index (LAI)

For estimating sodium and potassium, the procedure of Ahanger et al. (2015) was used. The Na^+^ and K^+^ contents were determined by flame photometry (Jenway ‐Flame Photometer Models PFP7). To estimate the area index, the leaf area meter (Delta‐T Devices Ltd.) was employed after using treatments in the end of each cutting time.

### 2.8 Statistical analysis

The procedure as type3 in MIXED method of SAS v. 9.4 (SAS Institute) was applied to analyze data. The significant effects of WDS, PUT, cutting time, and their two‐ and three‐way interactions were considered fixed effects, while years, replicates ×years and years ×WDS × PUT ×cutting time were considered chance effects. The PDIFF choice of least square means adjusted for the Tukey‐Kramer was applied for mean comparisons. The interactions among experimental factors were separated by slicing procedure. The significance of linear and quadratic regression models (*p* > .05) was examined with polynomial orthogonal contrasts. Pearson's correlation coefficients were specified applying the CORR method.

## 3 RESULTS

### 3.1 Dry weight, LAI, and endogenous putrescine

**FIGURE 1:**
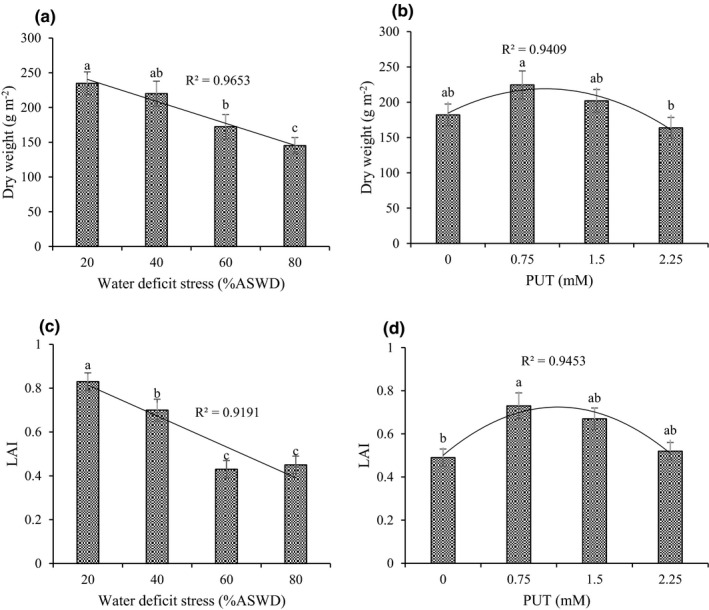
Main effect of water‐deficit stress and PUT on dry weight (a, b) and LAI (c, d). The different letters show significant difference at the level of 0.05. The error bars represent standard error

Based on the results analysis of variance, effects of water deficit, PUT, and cutting time were significant for the dry weight, LAI, and endo PUT of sage (Supplementary Information 2). Also, dry weight and LAI were higher in cutting 2 than in cutting 1. Endo PUT was higher in cutting 1 than in cutting 2 (Table 1). The maximum dry weight (234.76 g/m^2^) and LAI (0.83) were obtained in 20% ASWD. Indeed, there was a descending trend in dry weight and LAI by incrementing severity of water‐deficit stress (WDS) (Figure 1a,c). The response of dry weight and LAI to PUT can be expressed with a quadratic model, indicating that dry weight and LAI would reach their maximum (219.04 g/m^2^ and 0.72) under 0.98 and 1.14 mM of PUT, respectively (Figure 1b,d). There was an incrementing trend in endo PUT with increasing concentration of PUT. The maximum endo PUT (195.55 nmol/g FW) was obtained under 2.25 mM of PUT (Supplementary Information 3).

**TABLE 1:** Main effect of cutting time on dry weight, leaf area index (LAI), endogenous PUT (Endo PUT), essential oil (EO) content, essential oil (EO) yield, Fv/Fm, NPQ, photosynthetic rate (Pn), stomatal conductance (Gs), spad, chlorophyll a (Chl a), chlorophyll t (Chl t), Carotenoid, Na, K, and K/Na of *Salvia officinalis*

Some dependent traits	Cutting time	
	Cutting 1	Cutting 2
Dry weight (g/m^2^)	101.56b	284.53a
LAI	0.5b	0.7a
Endo PUT (nmol/g FW)	224.43a	64.32b
EO content (%)	1.37a	1.15b
EO yield (g/m^2^)	135.76b	323.69a
Fv/Fm	0.48b	0.74a
NPQ	0.31b	0.81a
Pn (μmol/m^2^ s^−1^)	14.88a	11.05b
Gs (mmol/m^2^ s^−1^)	0.05a	0.03b
Chl a (mg g^−1^ FW)	0.08a	0.06b
Chlo t (mg g^−1^ FW)	0.17a	0.14b
Carotenoid (mg g^−1^ FW)	0.77a	0.63b
Na (mg/g DM)	12.96b	16.81a
K (mg/g DM)	94.78a	67.02b
K/Na	7.98a	4.24b

### 3.2 Essential oil content and yield

**FIGURE 2:**
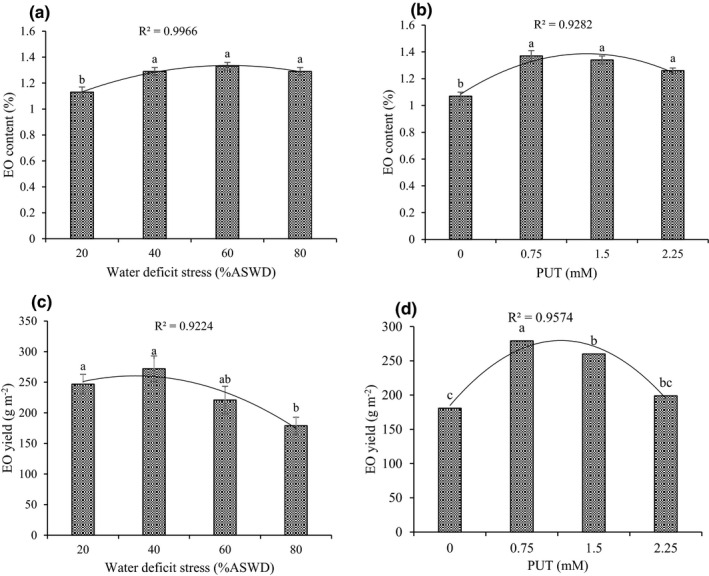
Main effect of water‐deficit stress and PUT on essential oil (EO) content (a, b) and EO yield (c, d). The different letters show significant difference at the level of 0.05. The error bars represent standard error

Based on the analysis of variance, the main effects of WDS, PUT, and cutting time were on EO content and EO yield (Supplementary Information 2). The EO content and yield were higher in cutting 1 and cutting 2, respectively (Table 1). The response of EO content and EO yield to WDS can be expressed by a quadratic model, showing that the EO content and EO yield would reach their maximum (1.45% and 260.47 g/m^2^) under 75.5% and 34.86% ASWD, respectively (Figure 2a,c). The response of EO content and EO yield to foliar application of PUT can be expressed with a significant quadratic model (*p* ≤ .01), suggesting that EO content and EO yield would reach their maximum (1.39% and 279.62 g/m^2^) under 1.34 and 1.16 mM of PUT (Figure 2b,d).

### 3.3 Photosynthetic gas exchange parameters, chlorophyll fluorescence, and greenness index

**FIGURE 3:**
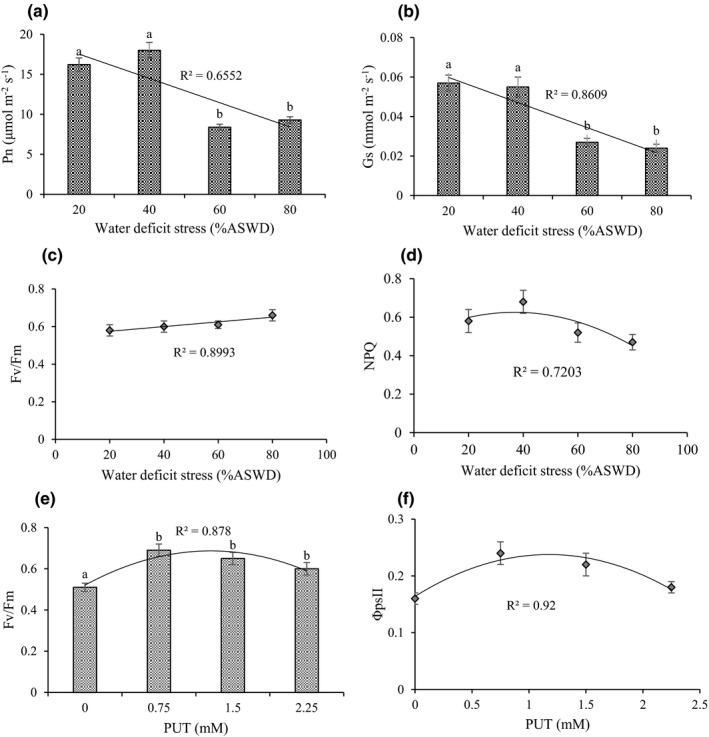
Main effect of water‐deficit stress on Pn (a), Gs (b), Fv/Fm (c), and NPQ (d) and the main effect of PUT on Fv/Fm (e) and ΦpsII (f). The different letters show significant difference at the level of 0.05. The error bars represent standard error

Based on the results analysis of variance, cutting time affected Pn, Gs, Fv/Fm, and NPQ significantly (Supplementary Information 2). Pn and Gs were higher in cutting 1 than in cutting 2. In contrast, Fv/Fm and NPQ were higher in cutting 2 than in cutting 1 (Table 1). The main effect of WDS was on Pn, Gs, and spad. Also, the main effect of PUT was in Fv/Fm and spad. None of the treatments was significantly different on qP (Supplementary Information 2). The maximum Pn (16.2 μmol/m^2^ s^‐1^) and Gs (0.057 mmol/m^2^ s^‐1^) were obtained in 20% ASWD. Indeed, there was a descending trend in Pn and Gs by incrementing severity of WDS (Figure 3a,b).

The response of Fv/Fm to WDS can be expressed with a linear model, indicating that Fv/Fm would reach its most by the increasing severity of WDS. A maximum Fv/Fm (0.66) was observed for 80% ASWD (Figure 3c). The response of NPQ and spad to WDS can be expressed by quadratic model, showing that NPQ and spad would attain their most (0.63 and 72.01) in 38.33% and 84.13% ASWD, respectively (Figure 3d and Supplementary Information 4). The response of Fv/Fm, ΦpsII, and spad to PUT can be expressed with a quadratic model, indicating that Fv/Fm, ΦpsII, and spad would reach their maximum (0.72, 0.24, and 56.1) under 1.27, 1.17, and 1.18 mM of PUT, respectively (Figure 3e,f, and Supplementary Information 5).

### 3.4 Chlorophyll *a, b*, total, and carotenoid

The result showed that the main influence of WDS and PUT was on Chl a, Chl b, Chl t, and carotenoid. Also, the significant effect of cutting time was on Chl a, Chl t, and carotenoid (Supplementary Information 2). Chl a, Chl t, and carotenoid were higher in cutting 1 than in cutting 2 (Table 1).

**FIGURE 4:**
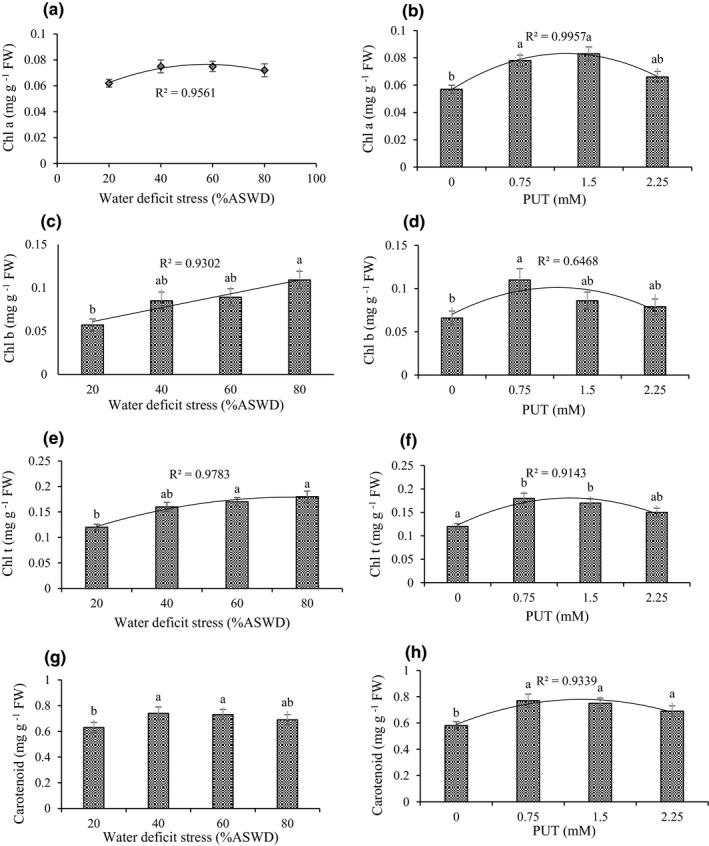
Main effect of water‐deficit stress and PUT on Chl a (a, b), Chl b (c, d), Chl t (e, f), and carotenoid (g, h). The different letters show significant difference at the level of 0.05. The error bars represent standard error

The response of Chl a and Chl t to WDS can be expressed by a quadratic model, showing that Chl a and Chl t would reach their maximum (0.08 and 0.17 mg g ^−1^ FW) in 60% and 70% ASWD, respectively (Figure 4a,e). The response of Chl b to WDS can be expressed with a linear model, indicating that Chl b would attain its most with an increasing severity of WDS. The maximum Chl b (0.11 mg g ^−1^ FW) was observed for 80% ASWD (Figure 4c). The highest carotenoid (0.63 and 0.74 mg g ^−1^ FW) was obtained in 20% and 40% ASWD, respectively (Figure 4g). The response of Chl a, Chl b, Chl t, and carotenoid to PUT can be expressed by a quadratic model, showing that Chl a, Chl b, Chl t, and carotenoid would reach their maximum (0.08, 0.10, 0.18, and 0.78 mg g ^−1^ FW) under 1.25, 1.17, 1.27, and 1.31 mM of PUT, respectively (Figure 4b,d,f,h).

### 3.5 Sodium, potassium, potassium/sodium ratio

Based on the results, that main effect of cutting time and PUT was significant for Na, K, and K/Na. Also, the main effect of WDS on Na and K (Supplementary Information 2). K and K/Na were higher in cutting 1 than in cutting 2. In contrast, Na was maximum in cutting 2 than in cutting 1 (Table 1).

**FIGURE 5:**
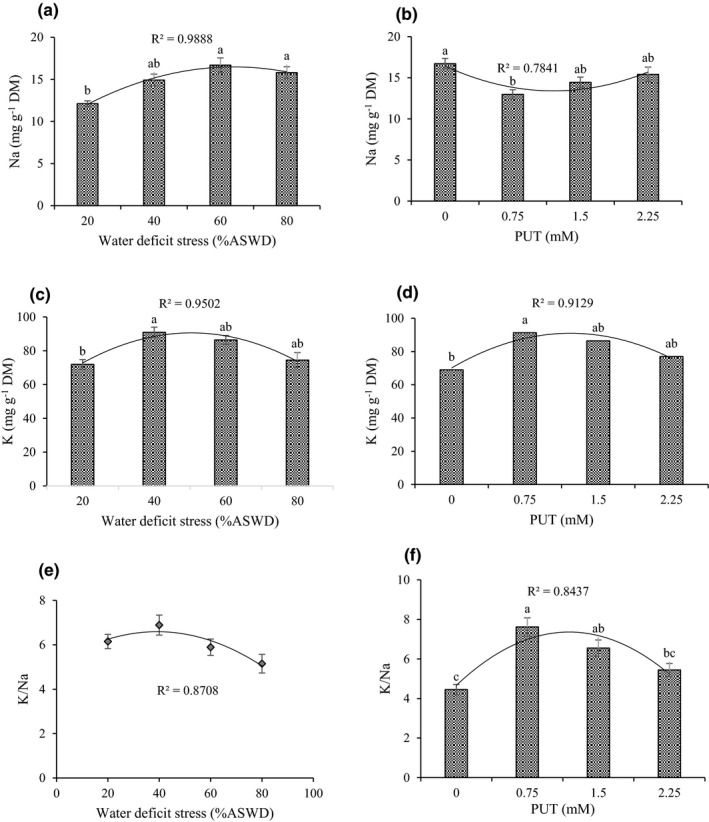
Main effect of water‐deficit stress and PUT on Na (a, b), K (c, d) and K/Na (e, f) contents. The different letters show significant difference at the level of 0.05. The error bars represent standard error

The response of Na to WDS and PUT can be expressed by a quadratic model, indicating that Na would attain its most (16.48 mg/g DM) under 63.96% ASWD and minimum (13.40 mg/g DM) under 1.20 mM of PUT, respectively (Figure 5a,b). The response of K and K/Na to WDS can be expressed with a quadratic model, suggesting that K and K/Na would reach their maximum (90.58 mg/g DM and 6.63) under 50.40% and 40.28% ASWD, respectively (Figure 5c,e). Also, the response of K and K/Na to PUT can be expressed by a quadratic model, showing that K and K/Na would reach their maximum (90.96 mg/g DM and 7.36) by applying 1.21 and 1.19 mM PUT, respectively (Figure 5d,f).

## 4 DISCUSSION

Slight water‐deficit stress (WDS) (34.86% ASWD) would be required for achieving the maximum EO content of sage. In the present study, the maximum EO yield of sage was produced under potential yield conditions in 75.5% ASWD. The lowest dry weight and LAI of sage were produced under 80% ASWD. Analogous to our results, the dry weight diminished with increasing WDS where the EO yield of sage was the highest under 60% ASWD (Govahi et al., 2015). Also, analogous to our results, Nowak et al. (2010) announced that the main part of monoterpenes increased, and biomass decreased in sage under WDS. Our results indicated that there was a high correlation between dry weight and LAI (0.72, *p* < .01) and EO yield (0.94, *p* < .01) (Supplementary Information 6). Also, dry weight, LAI, and EO yield were higher in cutting 2 than in cutting 1, in which these variations could be imputed to inter‐ and intraseasonal weather changes resulting from years and cutting times tested. Over both years, rainfall was higher by almost 0.64 mm in cutting 1 than in cutting 2, while temperature was lower by almost 13.47°C (Supplementary Information 1). The shift in EO yield suggests a possible corresponding shift and/or accumulation of EO in response to seasonal conditions. Indeed, the EO could increase, decrease, or disappear during cutting 1 and cutting 2. Thermoregulation is among the main causes, as the EO hydrophobic compounds could increase during the hot periods to protect the plant from desiccation (Kamatou et al., 2008). The genus salvia is one of the important known genera in the Labiatae family due to monoterpenes. Indeed, terpenes biosynthesis is composed of two separate ways, including methylerythritol 4‐phosphate (MEP) and mevalonate (MVA), occurring in plastids and cytoplasm in plants. As reviewed by Müller Verma and Shukla (2015), the MEP pathway is involved in the synthesis of carotenoids, isoprene, mono‐ and diterpenes, plant hormones, phytol, the side chain of Chl, tocopherols, phylloquinone, plastoquinones, etc. The polyamines in plants are found in cytoplasm and organelles such as mitochondria, chloroplasts, and vacuoles (Kusano et al., 2008). Based on the results, the highest dry weight, LAI, EO yield, and EO content were obtained under potential yield conditions with application of 0.98, 1.14, 1.16, and 1.34 mM of PUT, respectively. There was a high correlation between endo PUT and EO content (0.51, *p* < .01) and Gs (0.42, *p* < .05) (Supplementary Information 6). As an explanation, perhaps PUT enters the leaves by penetrating the cuticle or via the stomata, before entering the plant cell, where they can be practical in metabolism and are mainly carried to other portions via plasmodesmata. Hence, polyamines and monoterpenes were probably produced in the pathway of methylerythritol 4‐phosphate (MEP) and EO content affected by PUT.

In this study, the lowest LAI, Pn, and Gs were observed under 80% ASWD. Also, there was a high correlation among Pn and LAI (0.48, *p* < .01) and Gs (0.81, *p* < .01) (Supplementary Information 6). Probably, the minimum LAI resulted due to minimum of Pn under 80% ASWD. In addition, 80% ASWD‐induced reduction of Pn was accompanied by a reduction of Gs, suggesting that the effect of WDS on Pn can be due to stomatal agents (Yang & Lu, 2005). Based on our results, the levels of PUT were not accompanied by a considerable corresponding shift in Pn and Gs, indicating that stomatal guidance was not the only agent for PUT‐induced shifts in photosynthesis. Skowron et al. (2021) also reached similar results. The decreased photosynthesis can be due to nonstomatal limitations including the stagnation in PSII activity and electron transport (das Neves et al., 2008; Xia et al., 2004). Results showed that there was a negative correlation among NPQ and Gs (−0.37, *p* < .05), Fv/Fm and Pn (−0.38, *p* < .05), and Fv/Fm and Gs (−0.71, *p* < .01) (Supplementary Information 6). Indeed, there was a negative correlation among photosynthetic gas exchange parameter and chlorophyll fluorescence parameter.

Electron transfer in the photosynthesis process is necessary for assimilation. The catalytic protein PSII is the first component of the electron transport chain (Liao et al., 2013). Based on our results, the highest Fv/Fm was obtained under 80% ASWD. Also, Fv/Fm was higher in cutting 2 than in cutting 1. Indeed, elevation of Fv/Fm indicates the protection of light in sage as well as higher performance of PSII due to increase in electron transfer from PSI to PSII under WDS. Ranjbar Fordoei (2015) also reached similar results. The regression results indicated that greenness index and Chl a, Chl b, Chl t, and carotenoid increased with intensity of WDS. According to our results, Pirzad et al. (2011) reported that Chl increased under WDS. Light sorption through Chl can be separated into three portions: 1, imbibed light is transferred to a light reaction center for photochemical reaction (PSII photochemistry); 2, imbibed light energy is dissipated via heat radiation (thermal energy dissipation); and 3, imbibed light energy is dissipated via fluorescence radiation energy in PSII (NPQ) (Krause & Weis, 1991). Regression results showed that the NPQ increased under 38.33% ASWD. There was a negative correlation among NPQ and K (−0.51, *p* < .01) and K/Na (−0.59, *p* < .01) (Supplementary Information 6). Also, there were a positive correlation among NPQ and Na (0.48, *p* < .01) (Supplementary Information 6). In addition, NPQ and Na were higher in cutting 2 than in cutting 1. As a result, environmental stress makes the electron transfer chain saturated and leads to protons accumulating, whereby NPQ would show increment (Porcar‐Castell et al., 2014). By increasing this value, the chloroplasts are more capable of reversing the effects of WDS, as these organelles can decompose the excess excitation energy (Li et al., 2014). There was a negative correlation among NPQ and Chl a (−0.59, *p* < .01), Chl b (−0.38, *p* < .05), Chl t (−0.50, *p* < .01), and carotenoid (−0.55, *p* < .01) (Supplementary Information 6). Hence, it could be concluded that excess excitation energy is mostly used to produce Chl and carotenoids compared to the production of NPQ in WDS conditions in sage. There was a negative correlation among NPQ and EO content (−0.56, *p* < .01) (Supplementary Information 6). Also, NPQ and EO were higher in cutting 2 and cutting 1, respectively. Indeed, the synthesis of secondary metabolites and the biosynthesis of highly decreased compounds like isoprene are mainly involved in the waste of surplus photosynthetic energy (Wilhelm et al., 2011). Fv/Fm, ΦPSII, and spad exhibited a shift in pattern analogous to that of PUT, showing their most important effects on incrementing Fv/Fm, ΦPSII, and spad at concentrations of 1.27, 1.17, and 1.18, respectively. Indeed, Fv/Fm and ΦpsII improved after the PUT application; however, NPQ was not affected. These findings propose that, probably, the PUT improved the photosynthetic system by optimizing energy broadcast and by enhancing structure and function.

The maximum concentration of endo PUT was obtained by 2.25 mM of PUT. Based on our results, there was a high correlation between endo PUT and ΦpsII (0.55, *p* < .01) and Gs (0.42, *p* < .05) (Supplementary Information 6). This correlation could be ascribed to the role of PUT in stimulating plant growth by photosynthesis. There was a negative correlation between endo PUT and NPQ plus Fv/Fm (−0.64, −0.72, *p* < .01) (Supplementary Information 6). Similar results were obtained by Razzaq et al. (2014). In the present study, there was a high correlation among endo PUT and Chl a (0.58, *p* < .01) Chl t (0.43, *p* < .01), and carotenoid (0.60, *p* < .01) (Supplementary Information 6). Also, PUT, Chl a, Chl t, and carotenoid were higher in cutting 1 than in cutting 2. PUT incremented the accumulation of Chl a, b, t and carotenoid, indicating that PUT improved the transport of photosynthetic matter. Perhaps PUT prevents loss of Chl by maintaining the integrity of thylakoid membrane molecular composition. Indeed, the amino group of PUT (diamine aminotransferase) transforms into oxoglutaric acid (the precursor of Chl) (Askar & Treptow, 1986). Similar results were obtained by Gupta et al. (2012).

According to regression results, the maximum and minimum Na concentration was observed in 63.96% ASWD and 1.20 mM of PUT, respectively. Also, K and K/Na would reach their maximum by applying 1.21 and 1.19 mM of PUT, respectively. Instead, there were positive and negative correlations among endo PUT and Na (−0.43, *p* < .01), K (0.58, *p* < .01), and K/Na (0.59, *p* < .01) (Supplementary Information 6). There was a positive correlation among K/Na and Pn (0.59, *p* < .01) and Gs (0.51, *p* < .01) (Supplementary Information 6). Also, K, K/Na, Pn, and Gs were higher in cutting 1 than in cutting 2. Indeed, stomatal and photosynthetic activity improved with increasing K levels with PUT. According to our results, Iqbal and Ashraf (2005) reported that PUT could adjust ion homeostasis, sorption, and translocation of poisonous ions. Indeed, the regulating effect of PUT on the ion balance is due to the aggregated endo PUT rather than rivalry among cationic PUT and Na at the absorption status (Ndayiragije & Lutts, 2006).

## 5 CONCLUSION

The study indicated that the maximum EO yield of sage was produced under potential yield conditions in 75.5% ASWD. Greenness index, Chl, and carotenoid increased by the intensity of WDS. The lowest LAI, Pn, and Gs were observed under 80% ASWD. But, the highest Fv/Fm was obtained under 80% ASWD. The reduced photosynthesis can be due to stomatal factors and nonstomatal limitations including the stagnation in PSII activity and electron transport. Regression results showed that the NPQ increased under 38.33% ASWD. There was a negative correlation between NPQ and K and K/Na. Indeed, environmental stresses make the electron transfer chain saturated and increment proton accumulation, whereby NPQ would increase. There was a negative correlation between NPQ and Chl and carotenoid. So, it could be concluded that excess excitation energy is mostly used to produce Chl and carotenoids compared to the production of NPQ under WDS conditions in sage. There was a negative correlation between NPQ and EO content. Also, NPQ and EO were higher in cutting 2 and cutting 1, respectively. Nonetheless, the dissipation of surplus photosynthetic energy is mainly accomplished by the biosynthesis of highly reduced compounds such as isoprene and secondary metabolites. PUT showed that Fv/Fm and ΦpsII improvement, however, did not affect NPQ. These findings propose that, probably, PUT optimized energy broadcast and improved the structure and function of the photosynthetic system, and thereby it can be promoting primary electron transfer in PSII. PUT incremented the accumulation of Chl a, Chl b, Chl t, and carotenoid, indicating that PUT improved the transport of the photosynthetic matter. The maximum concentration of endo PUT was obtained with 2.25 mM of PUT. There were positive correlations between K/N and endo PUT, Pn, and Gs. Indeed, stomatal and photosynthetic activity improved with increasing K levels with the application of PUT.

## Supporting information

App S1

## Data Availability

The data that support the findings of this study are available from the corresponding author by reasonable request.

## References

[fsn32741-bib-0001] Ahanger, M. A. , Agarwal, R. M. , Tomar, N. S. , & Shrivastava, M. (2015). Potassium induces positive changes in nitrogen metabolism and antioxidant system of oat (*Avena sativa* L. cultivar Kent). Journal of Plant Interactions, 10, 211–223.

[fsn32741-bib-0002] Alcázar, R. , Bitrián, M. , Bartels, D. , Koncz, C. , Altabella, T. , & Tiburcio, A. F. (2011). Polyamine metabolic canalization in response to drought stress in Arabidopsis and the resurrection plant *Craterostigma plantagineum* . Plant Signaling & Behavior, 6, 243–250. 21330782 10.4161/psb.6.2.14317 PMC3121985

[fsn32741-bib-0003] Alcázar, R. , Planas, J. , Saxena, T. , Zarza, X. , Bortolotti, C. , Cuevas, J. , Bitrián, M. , Tiburcio, A. F. , & Altabella, T. (2010). Putrescine accumulation confers drought tolerance in transgenic Arabidopsis plants over‐expressing the homologous Arginine decarboxylase 2 gene. Plant Physiology and Biochemistry, 48, 547–552. 10.1016/j.plaphy.2010.02.002 20206537

[fsn32741-bib-0004] Aloisi, I. , Cai, G. , Serafini‐Fracassini, D. , & Del Duca, S. (2016). Polyamines in pollen: From microsporogenesis to fertilization. Frontiers Plant Science, 7, 155. 10.3389/fpls.2016.00155 PMC4757701 26925074

[fsn32741-bib-0005] Altindal, D. , & Altindal, N. (2016). Sage (*Salvia officinalis*) Oils. Book of essential oils in food preservation. Flavor and Safety, 81, 715–721.

[fsn32741-bib-0006] Anjum, S. A. , Xie, Y. , Wang, L. C. , Saleem, M. F. , Man, C. , & Lei, W. (2011). Morphological, physiological and biochemical responses of plants to drought stress. African Journal of. Agricultural Research, 6, 2026–2032.

[fsn32741-bib-0007] Ashraf, M. , & Harris, P. J. C. (2013). Photosynthesis under stressful environments: An overview. Photosynthetica, 51, 163–190. 10.1007/s11099-013-0021-6

[fsn32741-bib-0008] Askar, A. , & Treptow, H. (1986). Biogene Amine in Lebensmitteln — Vorkommen, Bedeutung und Bestimmung. 197 Seiten, 34 Abb., 47 Tab. Verlag Eugen Ulmer. Stuttgart. Preis: 58, — DM. Food / Nahrung, 32, 420.

[fsn32741-bib-0009] Bahreininejad, B. , Razmjooa, J. , & Mirzab, M. J. (2013). Influence of water stress on moroho‐physiological and phytochemical traits in *Thymus daenensis* . International Journal of Plant Production, 7, 151–166.

[fsn32741-bib-0010] Bettaieb, I. , Knioua, S. , Hamrouni, I. , Limam, F. , & Marzouk, B. (2011). Water‐deficit impact on fatty acid and essential oil composition and antioxidant activities of cumin (*Cuminum cyminum* L.) aerial parts. Journal of Agricultural and Food Chemistry, 59, 328–334. 10.1021/jf1037618 21141890

[fsn32741-bib-0011] Bouchereau, A. , Aziz, A. , Larher, F. , & Martin‐Tanguy, J. (1999). Polyamines and environmental challenges: Recent development. International Journal of Plant Sciences, 140, 103–125. 10.1016/S0168-9452(98)00218-0

[fsn32741-bib-0012] das Neves, J. P. C. , Ferreira, L. F. P. , Vaz, M. M. , & Gazarini, L. C. (2008). Gas exchange in the salt marsh species atriplex portulacoides L. and *Limoniastrum monopetalum* L. in southern Portugal. Acta Physiologiae Plantarum, 30, 91–97. 10.1007/s11738-007-0094-6

[fsn32741-bib-0013] Emami Bistgani, Z. , Siadat, S. A. , Bakhshandeh, A. , Ghasemi Pirbalouti, A. , & Hashemi, M. (2017). Interactive effects of drought stress and chitosan application on physiological characteristics and essential oil yield of *Thymus daenensis* Celak. Crop Journal, 5, 407–415. 10.1016/j.cj.2017.04.003

[fsn32741-bib-0014] Gitelson, A. A. , Gritz, Y. , & Merzlyak, M. N. (2003). Relationships between leaf chlorophyll content and spectral reflectance and algorithms for non‐destructive chlorophyll assessment in higher plant leaves. Journal of Plant Physiology, 160, 271–282. 10.1078/0176-1617-00887 12749084

[fsn32741-bib-0015] Govahi, M. , Ghalavand, A. , Nadjafi, F. , & Sorooshzadeh, A. (2015). Comparing different soil fertility systems in Sage (*Salvia officinalis*) under water deficiency. Industrial Crops and Products, 74, 20–27. 10.1016/j.indcrop.2015.04.053

[fsn32741-bib-0016] Gupta, K. , Dey, A. , & Gupta, B. (2013). Plant polyamines in abiotic stress responses. Acta Physiologiae Plantarum, 35, 2015–2036. 10.1007/s11738-013-1239-4

[fsn32741-bib-0017] Gupta, S. , Agarwal, V. P. , & Gupta, N. K. (2012). Efficacy of putrescine and benzyladenine on photosynthesis and productivity in relation to drought tolerance in wheat (*Triticum aestivum* L.). Physiology and Molecular Biology of Plants, 18, 331–336. 10.1007/s12298-012-0123-9 24082495 PMC3550557

[fsn32741-bib-0018] Hazrati, S. , Tahmasebi‐Sarvestani, Z. , Modarres‐Sanavy, S. A. M. , Mokhtassi‐Bidgoli, A. , Mohammadi, H. , & Nicola, S. (2017). Effects of zeolite and water stress on growth: Yield and chemical compositions of *Aloe vera* L. Agricultural Water Management, 181, 66–72. 10.1016/j.agwat.2016.11.026

[fsn32741-bib-0019] Hazrati, S. , Tahmasebi‐Sarvestani, Z. , Modarres‐Sanavy, S. A. M. , Mokhtassi‐Bidgoli, A. , & Nicola, S. (2016). Effects of water stress and light intensity on chlorophyll fluorescence parameters and pigments of *Aloe vera* L. Plant Physiology and Biochemistry, 106, 141–148. 10.1016/j.plaphy.2016.04.046 27161580

[fsn32741-bib-0020] He, L. , Nada, K. , Kasukabe, Y. , & Tachibana, S. (2002). Enhanced susceptibility of photosynthesis to low‐temperature photoinhibition due to interruption of chill‐induced increase of S‐adenosylmethionine decarboxylase activity in leaves of spinach (*Spinacia oleracea* L.). Plant & Cell Physiology, 43, 196–206. 11867699 10.1093/pcp/pcf021

[fsn32741-bib-0021] Horton, P. , & Ruban, A. (2005). Molecular design of the photosystem II light‐harvesting antenna: Photosynthesis and photoprotection. Journal of Experimental Botany, 56, 365–373. 10.1093/jxb/eri023 15557295

[fsn32741-bib-0022] Iqbal, M. , & Ashraf, M. (2005). Changes in growth, photosynthetic capacity and ionic relations in spring wheat (*Triticum aestivum* L.) due to pre‐sowing seed treatment with polyamines. Plant Growth Regulation, 46, 19–30.

[fsn32741-bib-0023] Kamatou, G. P. P. , Van Zyl, R. L. , Van Vuuren, S. F. , Figueiredo, A. C. , Barroso, J. G. , Pedro, L. G. , & Viljoen, A. M. (2008). Seasonal variation in essential oil composition, oil toxicity and the biological activity of solvent extracts of three South African Salvia species. South African Journal of Botany, 74, 230–237. 10.1016/j.sajb.2007.08.002

[fsn32741-bib-0024] Koptur, S. (1985). Alternative defences against herbivores in Inga (Fabaceae: Mimosoideae) over an elevated gradient. Ecology, 66, 1639–1650.

[fsn32741-bib-0025] Krause, G. H. , & Weis, E. (1991). Chlorophyll fluorescence and photosynthesis: The basics. Annual Review of Plant Physiology, 42, 313–349. 10.1146/annurev.pp.42.060191.001525

[fsn32741-bib-0026] Kusano, T. , Berberich, T. , Tateda, C. , & Takahashi, Y. (2008). Polyamines: Essential factors for growth and survival. Planta, 228, 367–381. 10.1007/s00425-008-0772-7 18594857

[fsn32741-bib-0027] Kusano, T. , Yamaguchi, K. , Berberich, T. , & Takahashi, Y. (2007). The polyamine spermine rescues Arabidopsis from salinity and drought stresses. Plant Signaling & Behavior, 2, 251–252. 10.4161/psb.2.4.3866 19704669 PMC2634138

[fsn32741-bib-0028] Li, Q. , Deng, M. , Xiong, Y. , Coombes, A. , & Zhao, W. (2014). Morphological and photosynthetic response to high and low irradiance of *Aeschynanthus longicaulis* . The Scientific World Journal, 2014, 347461. 10.1155/2014/347461 25093201 PMC4100289

[fsn32741-bib-0029] Liao, J. , Xiao, X. , Song, Y. , Zhou, Q. , & Huang, Y. (2013). Effects of high temperature on grain‐filling of rice caryopsis and physiological and biochemical characteristic of flag leave at early milky stage. Chih Wu Sheng Li Hsueh T’ung Hsun, 49, 175–180.

[fsn32741-bib-0030] Lichtenthaler, H. K. (1987). Chlorophylls and carotenoids, the pigments of photosynthetic biomembranes. In: Douce, R. and Packer, L. (eds.), Metods Enzymol. 148, 350–382.

[fsn32741-bib-0031] Lütz, C. , Navakoudis, E. , Seidlitz, H. K. , & Kotzabasis, K. (2005). Simulated solar irradiation with enhanced UV‐B adjust plastid‐ and thylakoid‐associated polyamine changes for UV‐B protection. Biochimica Et Biophysica Acta, 1710, 24–33. 10.1016/j.bbabio.2005.09.001 16246296

[fsn32741-bib-0032] Maathuis, F. J. M. , & Amtmann, A. (1999). K+ nutrition and Na+ toxicity: The basis of cellular K+/Na+ ratios. Annals of Botany, 84, 123–133. 10.1006/anbo.1999.0912

[fsn32741-bib-0033] Maxwell, K. , & Johnson, G. N. (2000). Chlorophyll fluorescenceea practical guide. Journal of Experimental Botany, 51, 659–668. 10938857 10.1093/jxb/51.345.659

[fsn32741-bib-0034] Mohammadi, H. , Ghorbanpour, M. , & Brestic, M. (2018). Exogenous putrescine changes redox regulations and essential oil constituents in field‐grown *Thymus vulgaris* L. under well‐watered and drought stress conditions. Industrial Crops and Products, 122, 119–132. 10.1016/j.indcrop.2018.05.064

[fsn32741-bib-0035] Moseki, B. , & Dintwe, K. (2011). Effect of water stress on photosynthetic characteristics of two sorghum cultivars. African Journal of Plant Science, 5, 89–91.

[fsn32741-bib-0036] Müller, P. , Li, X. P. , & Niyogi, K. K. (2001). Non‐photochemical quenching. A response to excess light energy. Plant Physiology, 125, 1558–1566. 10.1104/pp.125.4.1558 11299337 PMC1539381

[fsn32741-bib-0037] Murchie, E. H. , & Lawson, T. (2013). Chlorophyll fluorescence analysis: A guide to good practice and understanding some new applications. Journal of Experimental Botany, 64, 3983–3998. 10.1093/jxb/ert208 23913954

[fsn32741-bib-0038] Nassar, A. H. , El‐Tarabily, K. A. , & Sivasithamparam, K. (2003). Growth promotion of bean (*Phaseolus vulgaris* L.) by a polyamineproducing isolate of Streptomyces griseoluteus. Plant Growth Regulation, 40, 97–106.

[fsn32741-bib-0039] Ndayiragije, A. , & Lutts, S. (2006). Do exogenous polyamines have an impact on the response of a salt‐sensitive rice cultivar to NaCl? Journal of Plant Physiology, 163(5), 506–516. 16473655 10.1016/j.jplph.2005.04.034

[fsn32741-bib-0040] Nowak, M. , Manderscheid, R. , Weigel, H. J. , Kleinwächter, M. , & Selmar, D. (2010). Drought stress increases the accumulation of monoterpenes in sage (*Salvia officinalis*), an effect that is compensated by elevated carbon dioxide concentration. Journal of Applied Botany and Food Quality, 83, 133–136.

[fsn32741-bib-0041] Paknejad, F. , Majidiheravan, E. , Noormohammadi, Q. , Siyadat, A. , & Vazan, S. (2007). Effects of drought stress on chlorophyll fluorescence parameters, chlorophyll content and grain yield of wheat cultivars. Research Journal of Biological Sciences, 7(6), 841–847. 10.3923/jbs.2007.841.847

[fsn32741-bib-0042] Pirzad, A. , Shakiba, M. R. , Zehtab‐Salmasi, S. , Mohammadi, S. A. , Arvishzadeh, R. , & Samadi, A. (2011). Effect of water stress on leaf relative water content, chlorophyll, proline and soluble carbohydrates in *Matricaria chamomilla* L. Journal of Medicinal Plants, 5, 2483–2488.

[fsn32741-bib-0043] Porcar‐Castell, A. , Tyystjärvi, E. , Atherton, J. , Van Der Tol, C. , Flexas, J. , Pfündel, E. E. , Moreno, J. , Frankenberg, C. , & Berry, J. A. (2014). Linking chlorophyll a fluorescence to photosynthesis for remote sensing applications: Mechanisms and challenges. Journal of Experimental Botany, 65, 4065–4095. 10.1093/jxb/eru191 24868038

[fsn32741-bib-0044] Ralph, P. J. , & Gademann, R. (2005). Rapid light curves: A powerful tool to assess photosynthetic activity. Aquatic Botany, 82, 222–237. 10.1016/j.aquabot.2005.02.006

[fsn32741-bib-0045] Ranjbar Fordoei, A. (2015). Variation characteristics of chlorophyll fluorescence of a typical eremophyte *Smirnovia Iranica* (Sabeti) during phenological stages in the sand drift desert (case study: In Kashan Region). Desert, 21, 35–41.

[fsn32741-bib-0046] Razzaq, K. , Sattar, A. , Ullah, A. , Shahid, M. , Ullah, A. , & Ullah, S. (2014). Role of putrescine in regulating fruit softening and antioxidative enzyme systems in ‘Samar Bahisht Chaunsa’ mango. Postharvest. Postharvest Biology and Technology, 96, 23–32. 10.1016/j.postharvbio.2014.05.003

[fsn32741-bib-0047] Roháček, K. (2002). Chlorophyll fluorescence parameters: The definitions, photosynthetic meaning, and mutual relationships. Photosynthetica, 40, 13–29. 10.1023/A:1020125719386

[fsn32741-bib-0048] Skowron, E. , & Trojak, M. (2021). Effect of exogenously‐applied abscisic acid, putrescine and hydrogen peroxide on drought tolerance of barley. Biologia, 76, 453–468. 10.2478/s11756-020-00644-2

[fsn32741-bib-0049] Souza, S. J. O. , de Ferri, P. H. , Fiuza, T. S. , Borges, L. L. , & Paula, J. R. (2018). Chemical composition and seasonality variability of the *Spiranthera odoratissima* volatile oils leaves. Revista Brasileira De Farmacognosia, 28(1), 16–20. 10.1016/j.bjp.2017.10.010

[fsn32741-bib-0050] Verma, N. , & Shukla, S. (2015). Impact of various factors responsible for fluctuation in plant secondary metabolites. Journal of Applied Research on Medicinal and Aromatic Plants, 2, 105–113. 10.1016/j.jarmap.2015.09.002

[fsn32741-bib-0051] Wilhelm, C. , & Selmar, D. (2011). Energy dissipation is an essential mechanism to sustain the viability of plants: The physiological limits of improved photosynthesis. Journal of Plant Physiology, 168, 79–87. 10.1016/j.jplph.2010.07.012 20800930

[fsn32741-bib-0052] Xia, J. R. , Li, Y. J. , & Zou, D. H. (2004). Effects of salinity stress on PSII in *Ulva lactuca* as probed by chlorophyll fluorescence measurements. Aquatic Botany, 80, 129–137. 10.1016/j.aquabot.2004.07.006

[fsn32741-bib-0053] Yang, X. H. , & Lu, C. M. (2005). Photosynthesis is improved by exogenous glycinebetaine in salt‐stressed maize plants. Plant Physiology, 124, 343–352. 10.1111/j.1399-3054.2005.00518.x

[fsn32741-bib-0054] Zandalinas, S. I. , Mittler, R. , Balfagón, D. , Arbona, V. , & Gómez‐Cadenas, A. (2017). Plant adaptations to the combination of drought and high temperatures. Journal of Plant Physiology, 162, 2–12. 10.1111/ppl.12540 28042678

[fsn32741-bib-0055] Zutic, I. , Putievsky, E. , & Dudai, N. (2014). Influence of harvest dynamics and cut height on yield components of sage (*Salvia officinalis* L.). Journal of Herbs, Spices & Medicinal Plants, 10, 49–61.

