# Supplementary material for: Effects of water‐deficit stress and putrescine on performances, photosynthetic gas exchange, and chlorophyll fluorescence parameters of Salvia officinalis in two cutting times
Source: Food Sci Nutr. 2022 Feb 26;10(5):1431–41. doi: 10.1002/fsn3.2741 (PMC9094464; doi:10.1002/fsn3.2741)
Supplement: Supplementary file 1 — App S1 [file FSN3-10-1431-s001.docx]

Appendix S1


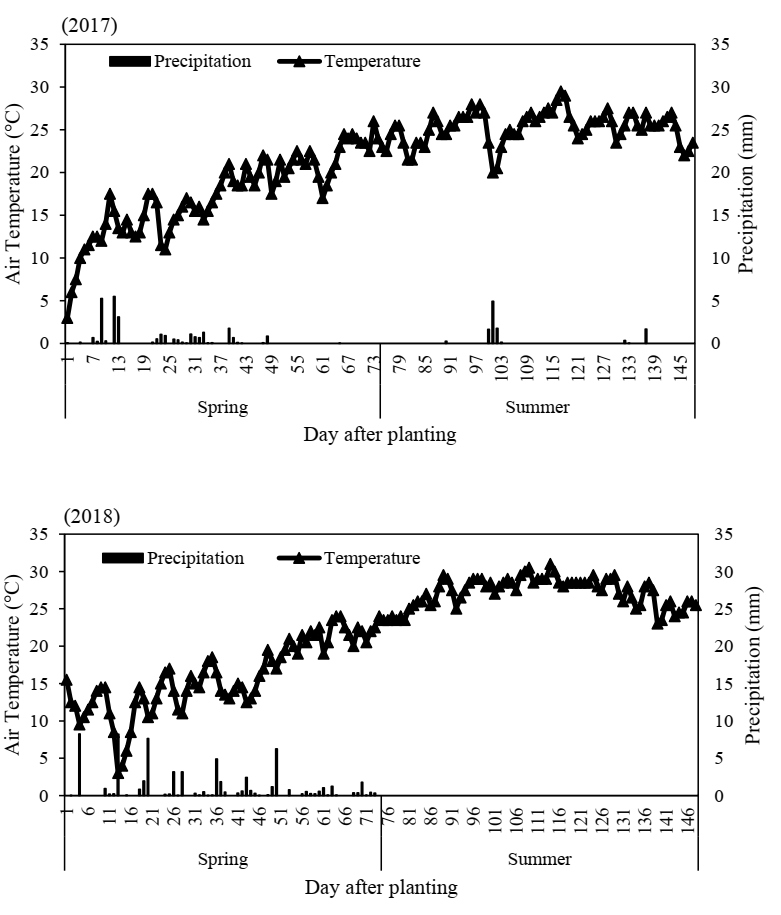
Supplementary Information 1. Daily average air temperatures (°C), and precipitation (mm) recorded during the growing season in 2017 and 2018.

Supplementary Information 2. Analysis of variance (mean square) and analysis of regression (mean square of linear and quadratic models) of dry weight, LAI, Endo PUT, EO content, EO yield, Fv/Fm, NPQ, ΦpsІІ, qP, Pn, stomatal conductance (Gs), spad, Chl a, Chl b, Chl t, Carotenoid, Na, K and K/Na of *Salvia officinalis* influenced by water deficit stress (D), PUT (P) and cutting time (C).

| Sources of variation | D | P | D×P | C | D×C | P×C | D×P×C | D-Linear | D-Quadratic | P-Linear | P-Quadratic |
| --- | --- | --- | --- | --- | --- | --- | --- | --- | --- | --- | --- |
| Num DF | 3 | 3 | 1 | 9 | 3 | 3 | 9 | 1 | 2 | 1 | 2 |
| Den DF | 31 | 31 | 31 | 31 | 31 | 31 | 31 | 190 | 189 | 190 | 189 |
| Dry weight (g m^-2^) | 9.18** | 3.64* | 1.04^ns^ | 177.64** | 1.92^ns^ | 0.70^ns^ | 1.09^ns^ | 19.51** | 9.79** | 1.08^ns^ | 3.52* |
| LAI | 11.66** | 4.06** | 0.75^ns^ | 11.71** | 0.60^ns^ | 0.22^ns^ | 0.49^ns^ | 52.74** | 28.32** | 0.03^ns^ | 8.32** |
| Endo PUT  (nmol g^-1^ FW) | 0.25^ns^ | 1.48^ns^ | 0.18^ns^ | 23.53** | 0.12^ns^ | 0.74^ns^ | 0.13^ns^ | 0.91^ns^ | 0.61^ns^ | 9.27** | 4.74** |
| EO content (%) | 6.32** | 14.36** | 0.30^ns^ | 39.86** | 2.01^ns^ | 1.69^ns^ | 0.85^ns^ | 12.08** | 11.23** | 13.50** | 27.74** |
| EO yield (g m^-2^) | 5.28** | 7.45** | 1.19^ns^ | 117.73** | 2.81^ns^ | 0.62^ns^ | 1.37^ns^ | 9.27** | 6.32** | 0.17^ns^ | 9.54** |
| Fv/Fm | 2.27^ns^ | 11.45** | 0.59^ns^ | 126.65** | 1.03^ns^ | 1.83^ns^ | 0.55^ns^ | 4.43* | 2.34^ns^ | 3.96* | 11.5** |
| NPQ | 2.08^ns^ | 0.49^ns^ | 0.49^ns^ | 65.37** | 1.08^ns^ | 0.27^ns^ | 0.94^ns^ | 4.2* | 3.07* | 0.24^ns^ | 0.95^ns^ |
| ΦpsІІ | 0.27^ns^ | 0.89^ns^ | 0.09^ns^ | 3.21^ns^ | 0.06^ns^ | 0.07^ns^ | 0.06^ns^ | 2.28^ns^ | 1.2^ns^ | 0.03^ns^ | 5.25** |
| qP | 0.81^ns^ | 0.44^ns^ | 0.37^ns^ | 0.14^ns^ | 0.06^ns^ | 0.64^ns^ | 0.35^ns^ | 2.09^ns^ | 2.07^ns^ | 0.09^ns^ | 0.31^ns^ |
| Pn (μmol m^-2^ s^-1^) | 13.97** | 1.13^ns^ | 0.17^ns^ | 8.78** | 0.42^ns^ | 0.01^ns^ | 0.06^ns^ | 73.04** | 36.53** | 0.48^ns^ | 2.39^ns^ |
| Gs (mmol m^-2^ s^-1^) | 14.00** | 1.12^ns^ | 0.17^ns^ | 35.35** | 2.15^ns^ | 0.11^ns^ | 0.18^ns^ | 66.89** | 33.29** | 0.83^ns^ | 2.3^ns^ |
| Spad | 10.62** | 20.72** | 0.56^ns^ | 0.07^ns^ | 0.84^ns^ | 0.41^ns^ | 0.28^ns^ | 45.03** | 24.38** | 1.94^ns^ | 51.88** |
| Chl a (mg g ^-1^ FW) | 1.74 | 5.82** | 0.45^ns^ | 15.93** | 0.07^ns^ | 0.78^ns^ | 0.25^ns^ | 2.61^ns^ | 3.29* | 2.66^ns^ | 12.35** |
| Chl b (mg g ^-1^ FW) | 4.52** | 3.45* | 0.58^ns^ | 3.18^ns^ | 0.39^ns^ | 0.58^ns^ | 0.46^ns^ | 12.2** | 6.13** | 0.13^ns^ | 3.15* |
| Chl t (mg g ^-1^ FW) | 6.31** | 6.18** | 1.35^ns^ | 13.96** | 1.01^ns^ | 1.05^ns^ | 0.41^ns^ | 21.37** | 11.71** | 12.4** | 10.44** |
| Car (mg g ^-1^ FW) | 3.93* | 10.13** | 0.57^ns^ | 27.84** | 0.10^ns^ | 0.99^ns^ | 0.23^ns^ | 0.84^ns^ | 2.11^ns^ | 2.42^ns^ | 5.65** |
| Na (mg g^-1^ DM) | 5.17** | 3.28* | 0.24^ns^ | 19.56** | 2.89^ns^ | 0.03^ns^ | 0.63^ns^ | 17.64** | 12.84** | 17.74** | 6.05** |
| K (mg g^-1^ DM) | 3.88* | 4.58** | 0.70^ns^ | 35.63** | 1.37^ns^ | 0.29^ns^ | 0.37^ns^ | 0.04^ns^ | 10.76** | 1.5^ns^ | 12.41** |
| K/Na | 2.34^ns^ | 8.48** | 0.60^ns^ | 57.14** | 0.26^ns^ | 0.98^ns^ | 0.23^ns^ | 4.95** | 4.25* | 1.19^ns^ | 16.74** |

ns: non-significant

*: α ≤ 0.05

**: α ≤ 0.01

Supplementary Information 3. Main effect of Exo PUT on Endo PUT. The different letters show signiﬁcantly different at the level of 0.05. The error bars represent standard error.

Supplementary Information 4. Main effect of water deficit stress on spad. The different letters show signiﬁcantly different at the level of 0.05. The error bars represent standard error.

Supplementary Information 5. Main effect of PUT on spad. The different letters show signiﬁcantly different at the level of 0.05. The error bars represent standard error.

Supplementary Information 6. Pearson’s correlation coefficients among dry weight, leaf area index (LAI), endogenous putrescine (endo put), essential oil (EO) content, essential oil (EO) yield, Fv/Fm, NPQ, ΦpsІІ, qP, photosynthetic rate (Pn), stomatal conductance (Gs), spad, chlorophyll a (Chl a), chlorophyll b (Chl b), chlorophyll t (Chl t), Carotenoid (Car), Na, K and K/Na of *Salvia officinalis* influenced by water deficit stress and putrescine.

|  | Dry weight | LAI | Endo put | EO content (%) | EO yield | Fv/Fm | NPQ | ΦpsІІ | qP | Pn | Gs | spad | Chl a | Chl b | Chlo t | Car | Na | K | K/Na |
| --- | --- | --- | --- | --- | --- | --- | --- | --- | --- | --- | --- | --- | --- | --- | --- | --- | --- | --- | --- |
| Dry weight | 1 |  |  |  |  |  |  |  |  |  |  |  |  |  |  |  |  |  |  |
| LAI | 0.72** | 1 |  |  |  |  |  |  |  |  |  |  |  |  |  |  |  |  |  |
| Endogenous put | -0.74** | -0.42* | 1 |  |  |  |  |  |  |  |  |  |  |  |  |  |  |  |  |
| EO content (%) | -0.52** | -0.24^ns^ | 0.51** | 1 |  |  |  |  |  |  |  |  |  |  |  |  |  |  |  |
| EO yield | 0.94** | 0.67** | -0.67** | -0.25^ns^ | 1 |  |  |  |  |  |  |  |  |  |  |  |  |  |  |
| Fv/Fm | 0.71** | 0.38* | -0.64** | -0.21^ns^ | 0.73** | 1 |  |  |  |  |  |  |  |  |  |  |  |  |  |
| NPQ | 0.79** | 0.45* | -0.72** | -0.56** | 0.71** | 0.68** | 1 |  |  |  |  |  |  |  |  |  |  |  |  |
| ΦpsІІ | -0.52** | -0.19^ns^ | 0.55** | 0.67** | -0.42* | -0.21^ns^ | -0.67** | 1 |  |  |  |  |  |  |  |  |  |  |  |
| qP | 0.16^ns^ | 0.13^ns^ | -0.21^ns^ | -0.19^ns^ | 0.15^ns^ | -0.06^ns^ | 0.23^ns^ | -0.34^ns^ | 1 |  |  |  |  |  |  |  |  |  |  |
| Pn | -0.02^ns^ | 0.48** | 0.26^ns^ | 0.17^ns^ | 0.00^ns^ | -0.38* | -0.18^ns^ | 0.11^ns^ | 0.22^ns^ | 1 |  |  |  |  |  |  |  |  |  |
| Gs | -0.38* | 0.10^ns^ | 0.42* | 0.10^ns^ | -0.40* | -0.71** | -0.37* | 0.09^ns^ | 0.21^ns^ | 0.81** | 1 |  |  |  |  |  |  |  |  |
| spad | -0.03^ns^ | 0.03^ns^ | 0.04^ns^ | 0.58** | 0.13^ns^ | 0.43* | -0.12^ns^ | 0.59** | -0.18^ns^ | -0.13^ns^ | -0.44* | 1 |  |  |  |  |  |  |  |
| Chl a | -0.47** | -0.18^ns^ | 0.58** | 0.76** | -0.31^ns^ | -0.24^ns^ | -0.59** | 0.74** | -0.14^ns^ | 0.22^ns^ | 0.15^ns^ | 0.59** | 1 |  |  |  |  |  |  |
| Chl b | -0.31^ns^ | -0.23^ns^ | 0.29^ns^ | 0.65** | -0.13^ns^ | 0.08^ns^ | -0.38* | 0.55** | -0.38* | -0.08^ns^ | -0.24^ns^ | 0.74** | 0.54** | 1 |  |  |  |  |  |
| Chl t | -0.41* | -0.25^ns^ | 0.43* | 0.76** | -0.23^ns^ | -0.08^ns^ | -0.50** | 0.65** | -0.29^ns^ | 0.01^ns^ | -0.11^ns^ | 0.72** | 0.74** | 0.94** | 1 |  |  |  |  |
| Car | -0.43* | -0.11^ns^ | 0.60** | 0.82** | -0.24^ns^ | -0.20^ns^ | -0.55** | 0.73** | -0.19^ns^ | 0.27^ns^ | 0.15^ns^ | 0.64** | 0.90** | 0.61** | 0.76** | 1 |  |  |  |
| Na | 0.28^ns^ | -0.23^ns^ | -0.43* | -0.29^ns^ | 0.29^ns^ | 0.38* | 0.48** | -0.50** | 0.10^ns^ | -0.56** | -0.54** | -0.08^ns^ | -0.39* | -0.16^ns^ | -0.26^ns^ | -0.43* | 1 |  |  |
| K | -0.50** | -0.11^ns^ | 0.58** | 0.73** | -0.35* | -0.36* | -0.51** | 0.66** | -0.01^ns^ | 0.41* | 0.33^ns^ | 0.47** | 0.74** | 0.39* | 0.53** | 0.83** | -0.36* | 1 |  |
| K/Na | -0.50** | 0.03^ns^ | 0.59** | 0.71** | -0.39* | -0.46** | -0.59** | 0.71** | -0.06^ns^ | 0.59** | 0.51** | 0.37* | 0.72** | 0.36* | 0.52** | 0.77** | -0.71** | 0.89** | 1 |

ns: non-significant

*: α ≤ 0.05

**: α ≤ 0.01
